# Canagliflozin, an SGLT2 inhibitor, attenuates the development of hepatocellular carcinoma in a mouse model of human NASH

**DOI:** 10.1038/s41598-018-19658-7

**Published:** 2018-02-05

**Authors:** Kumiko Shiba, Kyoichiro Tsuchiya, Chikara Komiya, Yasutaka Miyachi, Kentaro Mori, Noriko Shimazu, Shinobu Yamaguchi, Naomi Ogasawara, Makoto Katoh, Michiko Itoh, Takayoshi Suganami, Yoshihiro Ogawa

**Affiliations:** 1 Department of Molecular Endocrinology and Metabolism, Graduate School of Medical and Dental Sciences, Tokyo Medical and Dental University, Tokyo, Japan; 2 Department of Diabetes, Yamanashi-Kosei Hospital, Yamanashi, Japan; 3 Medical Science Department Ikuyaku, Integrated Value Development Division, Mitsubishi Tanabe Pharma Corporation, Tokyo, Japan; 4 Department of Organ Network and Metabolism, Graduate School of Medical and Dental Sciences, Tokyo Medical and Dental University, Tokyo, Japan; 5 Department of Molecular Medicine and Metabolism, Research Institute of Environmental Medicine, Nagoya University, Nagoya, Japan; 6 Department of Molecular and Cellular Metabolism, Graduate School of Medical and Dental Sciences, Tokyo Medical and Dental University, Tokyo, Japan; 7 Department of Medical and Bioregulatory Science, Graduate School of Medical Sciences, Kyushu University, Fukuoka, Japan; 8 Japan Agency for Medical Research and Development, CREST, Tokyo, Japan

## Abstract

Sodium glucose cotransporter 2 (SGLT2) inhibitors, an antidiabetic drug, promotes urinary excretion of glucose by blocking its reabsorption in the renal proximal tubules. It is unclear whether SGLT2 inhibition could attenuate nonalcoholic steatohepatitis (NASH) and NASH-associated hepatocellular carcinoma. We examined the preventive effects of an SGLT2 inhibitor canagliflozin (CANA) in Western diet (WD)-fed melanocortin 4 receptor-deficient (MC4R-KO) mice, a mouse model of human NASH. An eight-week CANA treatment attenuated hepatic steatosis in WD-fed MC4R-KO mice, with increased epididymal fat mass without inflammatory changes. CANA treatment for 20 weeks inhibited the development of hepatic fibrosis in WD-fed MC4R-KO mice. After one year of CANA treatment, the number of liver tumors was significantly reduced in WD-fed MC4R-KO mice. In adipose tissue, CANA suppressed the ratio of oxidative to reduced forms of glutathiones (GSSG/GSH) in WD-fed MC4R-KO mice. Treatment with GSH significantly attenuated the H_2_O_2_-induced upregulation of genes related to NADPH oxidase in 3T3-L1 adipocytes, and that of *Il6*, *Tgfb*, and *Pdgfb* in RAW264.7 cells. This study provides evidence that SGLT2 inhibitors represent the unique class of drugs that can attenuate or delay the onset of NASH and eventually hepatocellular carcinoma, at least partly, through “healthy adipose expansion”.

## Introduction

In recent decades, metabolic syndrome has become increasingly prevalent, with an increased incidence of nonalcoholic fatty liver disease (NAFLD)^1,2^. NAFLD is a clinical and pathologic term describing a disease spectrum ranging from simple steatosis to nonalcoholic steatohepatitis (NASH), cirrhosis, and hepatocellular carcinoma^3^. Obesity and type 2 diabetes mellitus (T2DM) are recognized as important risk factors for NAFLD; the prevalence of NAFLD is 4.6-times higher in the obese population than in normal individuals^4^, and 33–50% of T2DM patients have NAFLD^5^. Additionally, DM is moderately associated with deaths from cancers of the liver^6^, suggesting the clinical significance of preventive intervention for NAFLD. The insulin-sensitizing agent pioglitazone^7^, GLP-1 receptor agonist liraglutide^8^, and farnesoid X receptor agonist obeticholic acid^9,10^ reportedly improve histological parameters in human NASH. However, their efficacy and safety in long-term studies and preventive effects on NASH-associated carcinogenesis have not been confirmed.

Sodium glucose cotransporter 2 (SGLT2) inhibitors are a new class of oral hypoglycemic agents that work by decreasing glucose reabsorption in the renal proximal tubules. The net effect of increased renal glucose excretion has dual effects of insulin-independent glycemic control and caloric loss. Various animal models of NAFLD treated with SGLT2 inhibitors have demonstrated a protective effect on steatosis, inflammation, and fibrosis^11–17^. We recently reported that the SGLT2 inhibitor ipragliflozin promotes fat accumulation in epididymal fat without deteriorating adipose inflammation and prevents ectopic fat accumulation in the liver^18^. This suggests that SGLT2 inhibitors prevent hepatic steatosis not only via its insulin-independent glucose-lowering effect and caloric loss but also via modulating energy homeostasis and balance in adipose and non-adipose tissues. However, whether SGLT2 inhibitors can be effective in an animal model that closely reflects the liver condition of human NASH has not been addressed thus far. Moreover, it remains unknown whether SGLT2 inhibitors can prevent NASH-associated hepatocellular carcinoma.

We have developed an experimental mouse model of NASH; melanocortin 4 receptor-deficient (MC4R-KO) mice fed a western diet (WD) develop a liver condition like human NASH, in association with obesity, insulin resistance, and dyslipidemia^19^. Using these mice, here we demonstrate that an SGLT2 inhibitor canagliflozin (CANA) attenuates the development of hepatocellular carcinoma, as well as hepatic steatosis, inflammation, and fibrosis. CANA induced adipose expansion without deteriorating inflammation or fibrosis, referred to as “healthy adipose expansion.” We also confirmed that CANA altered glutathione metabolism to reduce oxidative stress in adipose tissue. It suggests that CANA prevented ectopic fat accumulation in the liver via promoting healthy adipose expansion and inhibited the development of hepatic fibrosis and hepatocellular carcinoma. Our findings with the MC4R-KO mice suggest that SGLT2 inhibitors have a significant clinical impact on human NASH and NASH-associated hepatocellular carcinoma.

## Materials and Methods

Details and additional information of the MATERIALS AND METHODS are included in the Supplementary Information.

### Drugs and diets

CANA was synthesized at the Medicinal Chemistry Laboratory at Mitsubishi Tanabe Pharma Corporation (Toda-shi, Saitama, Japan) and was mixed at 0.03% (w/w) with WD (WD/CANA). The dose of CANA was determined based on a previous report showing that 4-week treatment of 30 mg/kg/day CANA significantly increased urinary glucose excretion and decreased blood glucose in diet-induced obese mice^20^. The average amount of CANA consumed during the study was 20–30 mg/kg/day. Standard diet (SD, CRF-1; 357 kcal/100 g, 14% energy as fat) and western diet (WD, D12327; 460 kcal/100 g, 40% energy as fat) were purchased from Oriental Yeast. Co. Ltd. (Tokyo, Japan) and Research Diets. Inc. (New Brunswick, NJ, USA), respectively. Food consumption was calculated by subtracting remaining food, including any spilled food in cages, from a weighed aliquot at indicated intervals. Energy intake was then calculated on the basis of diet formula.

### Animals and experimental protocol

The MC4R-KO mice on the C57BL/6J background were a gift from Dr. Joel K. Elmquist (University of Texas Southwestern Medical Center, Dallas, TX, USA)^21^. We obtained male C57BL/6J mice (controls) from CLEA Japan, Inc. (Tokyo, Japan). Both WT and MC4R-KO mice were fed a SD until 8-week-old with free access to water. After that, MC4R-KO mice were fed a WD or a WD/CANA for 8, 20, or 52 weeks. Wild-type mice were fed a SD throughout the experiments. Eight- and 20-week treatment protocols were conducted at the Medicinal Chemistry Laboratory at Mitsubishi Tanabe Pharma Corporation, and 52-week treatment protocol was conducted at the Tokyo Medical and Dental University. For measurement of blood glucose levels during CANA treatment, blood was obtained at indicated intervals by tail vein puncture from non-fasted conscious mice around 2 pm. After 8-, 20-, and 52-week of CANA treatment, blood samples and tissues were obtained by cardiac puncture around 10am from non-fasted mice under intraperitoneal pentobarbital anesthesia (60 mg/kg). Only for measurement of HOMA-R and Adipo-IR, blood samples were obtained after an 18-hour fast. Spot urine was collected in the fed state around 10am; the mouse was held at the dorsal position and urinated, and the urine was collected in the sterile petri dish. All the animals were housed under conventional conditions of temperature, humidity, and light (12 h light: 12 h dark). The Tokyo Medical and Dental University Committee on Animal Research and the Institutional Animal Care and Use Committee of Mitsubishi Tanabe Pharma Corporation approved the protocols and procedures used in this study. All methods involving animals were performed in accordance with the relevant guidelines and regulations.

### Biochemical assays

Blood glucose levels were measured using a glucometer (Glutest PRO R (Sanwa Kagaku Kenkyusho Co., Ltd., Aichi, Japan) for a 52-week treatment protocol, or Autokit Glucose C2 (Wako Pure Chemical Industries, Ltd., Osaka, Japan) for 8- and 20-week treatment protocols. Urine glucose and creatinine levels were analyzed with enzymatic assays in a laboratory of Oriental Yeast Co., Ltd. (Tokyo, Japan). Non-esterified fatty acid (NEFA) and triglyceride (TG) were measured using NEFA C-Test Wako (Wako Pure Chemical Industries, Ltd., Osaka, Japan) and TG E-Test Wako (Wako Pure Chemical Industries, Ltd., Osaka, Japan), respectively.

### Metabolomic analysis of epididymal adipose tissue

Epididymal fat from MC4R-KO mice fed a WD or WD/CANA for 8 weeks was used for metabolomic analysis (n = 7). Metabolomic analysis was performed at the Human Metabolome Technologies (HMT), Inc. (Tsuruoka, Japan).

### Cell culture

3T3-L1 pre-adipocytes and RAW 264.7 cells were purchased from ATCC. 3T3-L1 pre-adipocytes were grown and differentiated to adipocytes as previously described^22^. Fully differentiated adipocytes or RAW 264.7 cells were washed with PBS, and incubated in a serum-free DMEM containing 0.25% bovine serum albumin with or without 1 mM GSH ester (G1404, Sigma-Aldrich Japan, Tokyo, Japan). After 1 h, hydrogen peroxide (Wako Pure Chemical Industries, Ltd.) was added to the culture medium to attain a final concentration of 200 μM and incubated for 18 h.

### Quantitative RT-PCR

Total RNA was extracted from mice tissues using the Sepasol reagent (Nacalai Tesque, Inc., Kyoto, Japan) and performed the first-strand cDNA synthesis using ReverTra Ace (Toyobo Co., Ltd., Osaka, Japan) and Random Primer (Thermo Fisher Scientific, Inc., Waltham, MA, USA). Quantitative RT-PCR was performed using StepOnePlus Real-time PCR System with Fast SYBR Green Master Mix Reagent (Thermo Fisher Scientific, Inc.). Primers are listed in Table S1. The relative gene expression was determined using the comparative CT method and normalized to the 36B4 levels.

### Western blotting

The mice liver tumor tissues were homogenized in a lysis buffer (2% SDS, 4 M Urea, 1 mM EDTA, 150 mM NaCl, 50 mM Tris; pH 8.0), centrifuged, separated the samples using 8% SDS-PAGE, and transferred to PVDF membranes. Phospho (Ser473)-Akt (Cat. #9271, Cell Signaling Technology, Danvers, MA, USA) and a total Akt antibody (Cat. #9272, Cell Signaling Technology) were used for immunoblotting. Immunoblots were detected with ECL Prime Western Blotting Detection Reagent and ImageQuant LAS 4000 mini (GE Healthcare, Little Chalfont, UK) and quantified using NIH ImageJ software.

### Statistical analysis

All datasets were tested for Gaussian distribution using a Kolmogorov‐Smirnov test. For normally distributed values, statistical analysis was performed with the Student’s t test for unpaired observations, or analysis of variance (ANOVA) followed by Bonferroni post hoc test, and data are given as mean ± standard error of the mean (SEM). Repeated measure based parameters were analyzed using two-way ANOVA followed by Bonferroni post hoc test. For non-Gaussian distributed values, data are expressed as box-and-whisker plots with median values and 10, 25, 75, and 90 percentiles. Nonparametric statistical analysis was performed with the Mann‐Whitney or Kruskal‐Wallis test with Dunn post hoc test. In post hoc analyses, MC4R-KO/WD and MC4R-KO/WD/CANA, or H_2_O_2_ and H_2_O_2_ + GSH was pre-selected as a subset of means or medians to compare. Statistical analyses were performed using GraphPad PRISM, 6.0 (GraphPad Software, Inc., La Jolla, CA, USA). Differences were considered significant at *P* < 0.05.

## Results

### CANA improves hyperglycemia, hyperinsulinemia, and hepatic steatosis in WD-fed MC4R-KO mice

**Figure 1:**
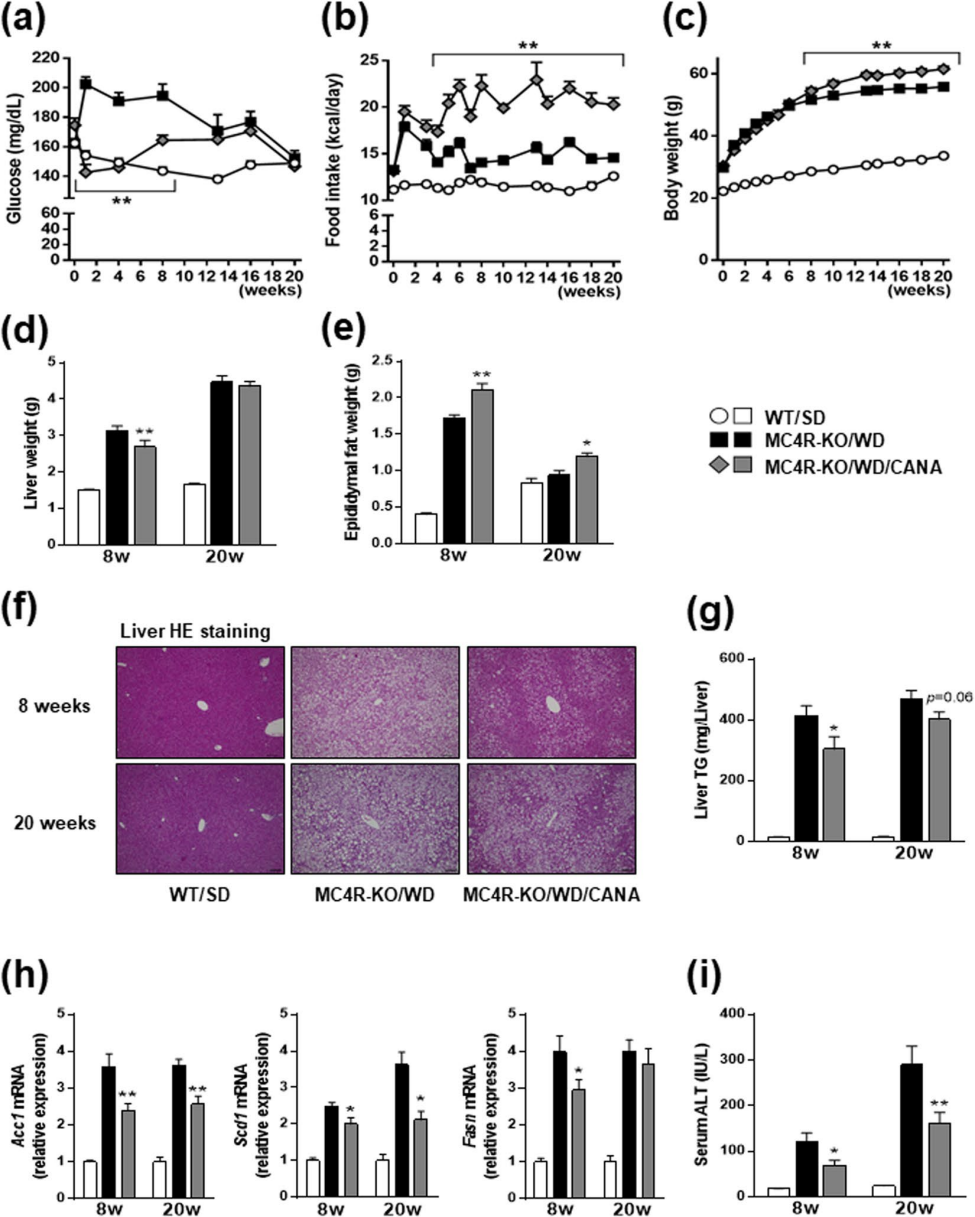
CANA attenuates hepatic steatosis in WD-fed MC4R-KO mice. The changes in (**a**) blood glucose level, (**b**) food intake, and (**c**) body weight during CANA treatment for 20 weeks. Weights of the (**d**) liver and (**e**) epididymal fat after 8 and 20 weeks of CANA treatment. (**f**) Hematoxylin and eosin (HE) staining and (**g**) triglyceride (TG) content of the liver. (**h**) Expression levels of lipogenesis-related genes in the liver. (**i**) Serum ALT levels. WT, wild type; SD, standard diet; WD, Western diet; CANA, canagliflozin. Original magnification, x200. Scale bars, 100 μm. *p < 0.05, **p < 0.01 vs MC4R-KO/WD. Statistical analyses were performed using two-way (**a**,**b** and **c**) or one-way ANOVA (**d**,**e**,**g**,**h** and **i**) followed by Bonferroni post hoc test; MC4R-KO/WD and MC4R-KO/WD/CANA was pre-selected as a subset of means to compare. *n* = 15.

We examined whether CANA inhibits the development of insulin resistance- and obesity-associated metabolic phenotypes in MC4R-KO mice on a WD feeding regimen. CANA significantly improved hyperglycemia and hyperinsulinemia with increased urinary excretion at 8 weeks of treatment in non-fasted MC4R-KO mice fed a WD (Fig. 1a, Table 1, and Supplementary Figure 1a). CANA significantly increased serum TG levels, but did not affect serum total- or active-GLP-1, or NEFA levels in non-fasted WD-fed MC4R-KO mice (Table 1). In fasted WD-fed MC4R-KO mice after 8 weeks of CANA treatment, hyperglycemia and hyperinsulinemia were markedly improved (Supplementary Figure 1b). Consequently, HOMA-R and Adipo-IR, indices of systemic and adipose insulin resistance^23,24^, respectively, were significantly reduced by CANA treatment (Supplementary Figure 1c). Four and eight weeks of CANA treatment increased their energy intake along with hyperphagia (Fig. 1b) and their body weight (Fig. 1c), respectively. Whereas eight weeks of CANA treatment significantly decreased liver weight (Fig. 1d), 8 and 20 weeks of CANA treatment increased epididymal fat weight in WD-fed MC4R-KO mice (Fig. 1e). CANA significantly attenuated hepatic lipid deposition and/or TG content (Fig. 1f and g) associated with a reduced expression of *de novo* lipogenic genes such as acetyl-CoA carboxylase 1 (*Acc1*), stearoyl-CoA desaturase 1 (*Scd1*), and fatty acid synthase (*Fasn*) in the liver of WD-fed MC4R-KO mice (Fig. 1h). Accordingly, CANA suppressed the elevation of serum ALT levels (Fig. 1i) and upregulated the expression of the gluconeogenic genes, glucose-6-phosphatase (*G6pc*) and phosphoenolpyruvate carboxykinase (*Pck1*), in WD-fed MC4R-KO mice (Supplementary Figure 2a).

**Table 1:** Metabolic parameters of non-fasted WD-fed MC4R-KO mice after CANA treatment for 8 and 20 weeks.

	8 weeks			20 weeks		
	WT/SD	MC4R-KO/WD	MC4R-KO/WD/CANA	WT/SD	MC4R-KO/WD	MC4R-KO/WD/CANA
Insulin (ng/ml)	0.81 ± 0.13	9.98 ± 1.00	6.28 ± 0.98**	1.59 ± 0.24	10.05 ± 0.43	10.78 ± 0.61
Glucagon (pg/ml)	6.10 ± 0.90	10.77 ± 1.58	10.29 ± 2.53	8.91 ± 1.89	24.07 ± 5.44	26.34 ± 3.27
tGLP-1 (pg/ml)	15.00 ± 4.26	25.57 ± 4.28	31.21 ± 4.04	ND	ND	ND
aGLP-1 (pg/ml)	ND	ND	ND	4.06 ± 1.29	5.82 ± 0.68	7.55 ± 2.00
TG (mg/dl)	83.07 ± 6.30	116.92 ± 10.29	160.96 ± 18.98*	77.87 ± 7.02	64.96 ± 5.29	84.15 ± 7.44
NEFA (mEq/L)	0.39 ± 0.02	0.55 ± 0.03	0.61 ± 0.04	0.45 ± 0.03	0.53 ± 0.03	0.57 ± 0.04

SD, standard diet; WD, western diet; CANA, Canagliflozin; tGLP-1, total GLP-1; aGLP-1, active GLP-1; TG, triglyceride; NEFA, non-esterified fatty acid. Data are mean ± SEM, *p < 0.05, **p < 0.01 vs MC4R-KO/WD; one-way ANOVA. *n* = 15.

SGLT1 gene (*Slc5a1*) was expressed in various organs such as the liver, lung, and pancreas (Supplementary Figure 2b). Among hepatocytes, Kupffer cells, and stellate cells, it was expressed mainly in hepatocytes. In contrast, SGLT2 gene (*Slc5a2*) was expressed mainly in the kidneys among the various organs (Supplementary Figure 2b). In the liver, SGLT1 protein was expressed in the biliary tract, and SGLT2 protein was expressed in the central vein and biliary tract (Supplementary Figure 2c).

### CANA inhibits hepatic inflammation and fibrosis in WD-fed MC4R-KO mice

**Figure 2:**
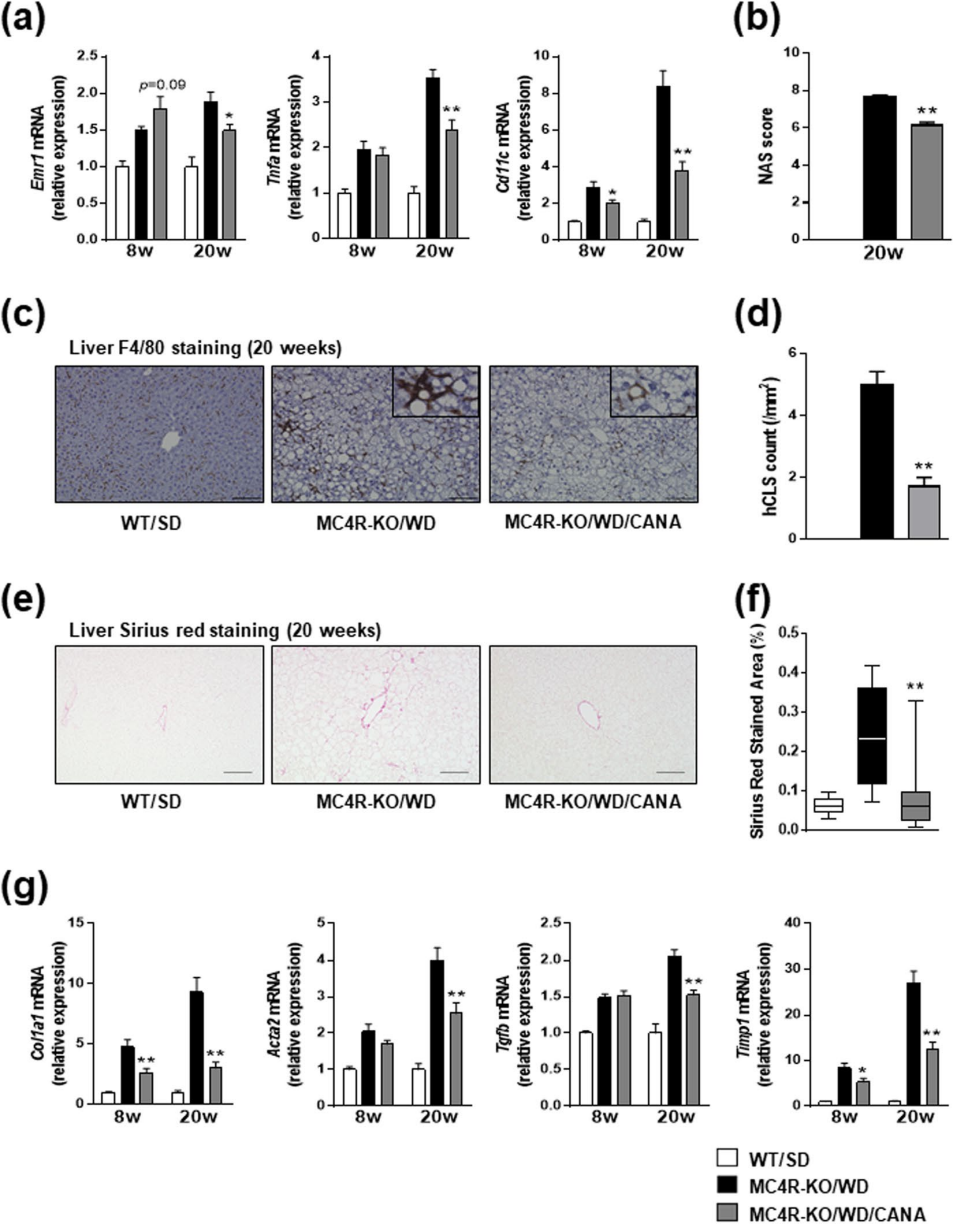
CANA inhibits hepatic inflammation and fibrosis in WD-fed MC4R-KO mice. (**a**) Expression levels of inflammation-related genes in the liver. (**b**) NAFLD activity score (NAS). (**c**) F4/80 immunohistochemical analysis of the liver. (**d**) Quantification of the number of hepatic crown-like structure (hCLS) in the liver. (**e**) Sirius red staining of the liver sections and (**f**) quantification. (**g**) Expression levels of fibrosis-related genes in the liver. Original magnification, ×200. Scale bars, 100 μm. *p < 0.05, **p < 0.01 vs MC4R-KO/WD. Statistical analyses were performed using one-way ANOVA (**a**,**b**,**d** and **g**) or Kruskal‐Wallis test (**e**) followed by Bonferroni or Dunn post hoc test, respectively; MC4R-KO/WD and MC4R-KO/WD/CANA was pre-selected as a subset of means or medians to compare. *n* = 15.

We next examined whether CANA prevents the development of hepatic inflammation and fibrosis in WD-fed MC4R-KO mice. Hepatic inflammation was demonstrated by an elevated expression of the common macrophage marker F4/80 gene (*Emr1*), which was inhibited by 20 weeks of CANA treatment (Fig. 2a). Twenty weeks of CANA treatment downregulated the gene expression of tumor-necrosis factor-α (*Tnfa*), and pro-inflammatory M1 macrophage marker *Cd11c* in the liver of WD-fed MC4R-KO mice, without affecting the anti-inflammatory M2 macrophage marker *Cd206* (data not shown). We histologically assessed the severity of liver injury with the NAFLD Activity Score (NAS); whereas 20 weeks of CANA treatment did not affect the steatosis score in WD-fed MC4R-KO mice (Supplementary Figure 3), the scores for lobular inflammation and hepatocyte ballooning were reduced by CANA treatment. As a result, CANA lowered the overall NAS in WD-fed MC4R-KO mice (Fig. 2b).

We previously reported the unique histological structure, hCLS, in the liver from MC4R-KO mice, where dead hepatocytes were surrounded by macrophages and suggested that hCLS serves as an origin of hepatic inflammation and fibrosis during the progression from simple steatosis to NASH^25^. In this study, MC4R-KO mice developed hCLS during the 20-week WD-feeding, as assessed by immunostaining for a macrophage marker F4/80, which was markedly reduced by CANA treatment (Fig. 2c and d). Consistent with this observation, 20 weeks of CANA treatment significantly reduced liver fibrosis in MC4R-KO mice (Fig. 2d and e), associated with the suppression of fibrogenic genes such as *Col1a1*, *Acta2*, *Tgfb1*, and *Timp1* (Fig. 2f).

### CANA reduces adipose tissue inflammation and fibrosis in WD-fed MC4R-KO mice

**Figure 3:**
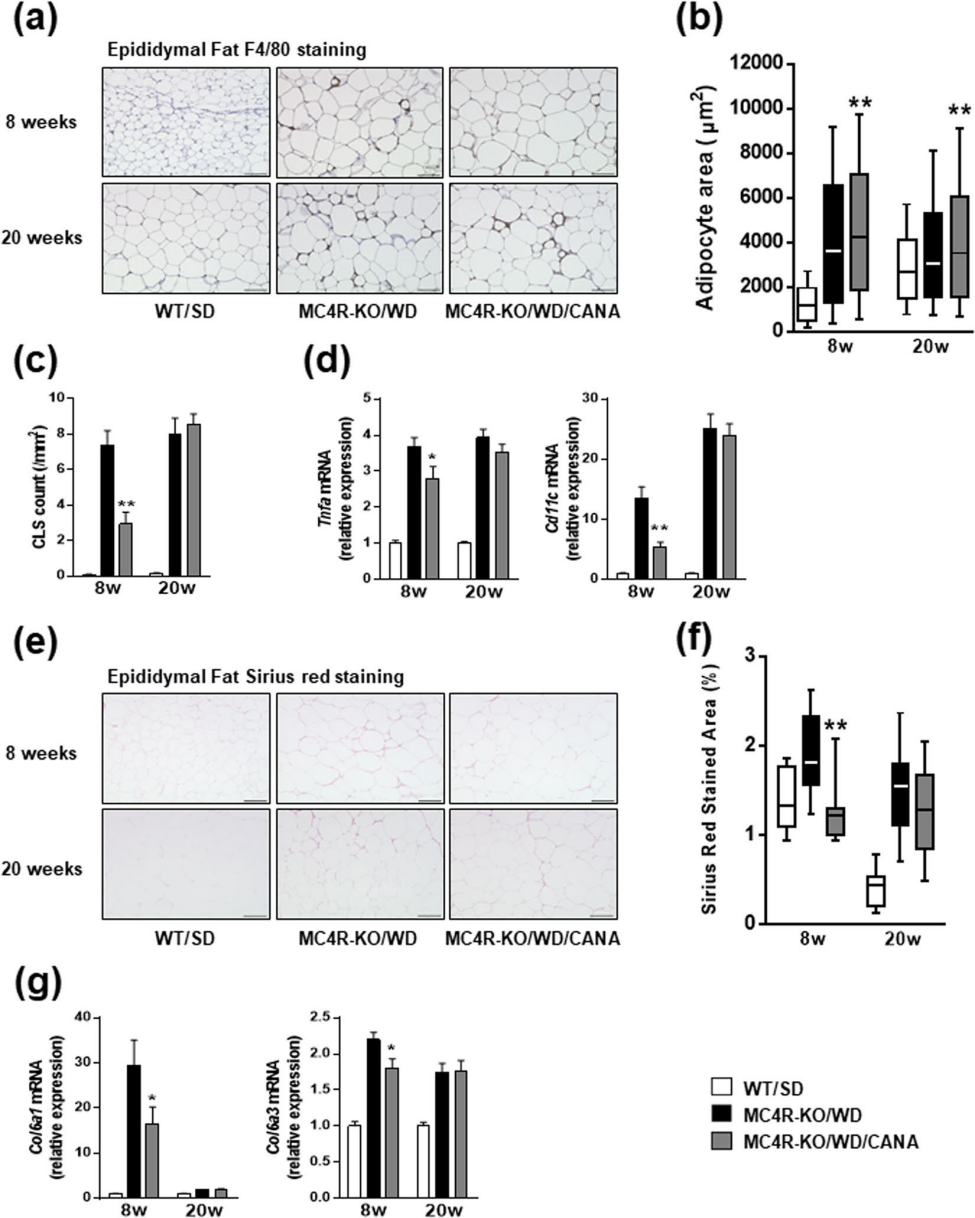
CANA reduces inflammation and fibrosis in adipose tissue of WD-fed MC4R-KO mice. (**a**) F4/80 immunohistochemical analysis of epididymal fat. (**b**) Adipocyte area in epididymal fat. (**c**) Quantification of the number of crown-like structure (CLS) in epididymal fat. (**d**) Expression levels of inflammation-related genes in epididymal fat. (**e**) Sirius red staining of epididymal fat and (**f**) quantification. (**g**) Expression levels of collagen genes in epididymal fat. Original magnification, ×200. Scale bars, 100 μm. *p < 0.05, **p < 0.01 vs MC4R-KO/WD. Statistical analyses were performed using Kruskal‐Wallis test (**b** and **f**) or one-way ANOVA (**c**,**d** and **g**) followed by Dunn or Bonferroni post hoc test, respectively; MC4R-KO/WD and MC4R-KO/WD/CANA was pre-selected as a subset of means or medians to compare. *n* = 15.

Consistent with the increase in epidydimal fat mass in CANA-treated mice, histological analysis revealed that CANA promoted adipocyte hypertrophy as assessed by cell size in WD-fed MC4R-KO mice (Fig. 3a and b). Despite adipocyte hypertrophy, the number of crown-like structures (CLS), where macrophages are considered to scavenge the residual lipid droplets of dead or dying adipocytes^26^, was markedly decreased by eight weeks of CANA treatment (Fig. 3c). Expression of *Tnfa* and *Cd11c* were upregulated in the epididymal fat of WD-fed MC4R-KO mice compared with SD-fed WT mice, which were inhibited by eight weeks of CANA treatment (Fig. 3d). In accordance with decreased number of CLS, eight weeks of CANA treatment significantly prevented WD-induced fibrosis in adipose tissue (Fig. 3e and f), associated with a reduced expression of *Col6a1* and *Col6a3* (Fig. 3g). Whereas expression of adipogenic (*Pparg* and *Cebpa*), and hypoxic/angiogenic genes (*Hif1a* and *Vegfa*) were unaffected by CANA (Supplementary Figure 4a), expression of brown adipocyte-selective genes (*Ucp1*, *Prdm16*, and *Cidea*) were suppressed by CANA in WD-fed MC4R-KO mice (Supplementary Figure 4b).

These data indicate that an increase in the epididymal fat mass in CANA-treated MC4R-KO mice does not deteriorate macrophage accumulation, inflammation, or fibrosis.

### CANA decreases oxidative stress in epididymal fat of WD-fed MC4R-KO mice

**Figure 4:**
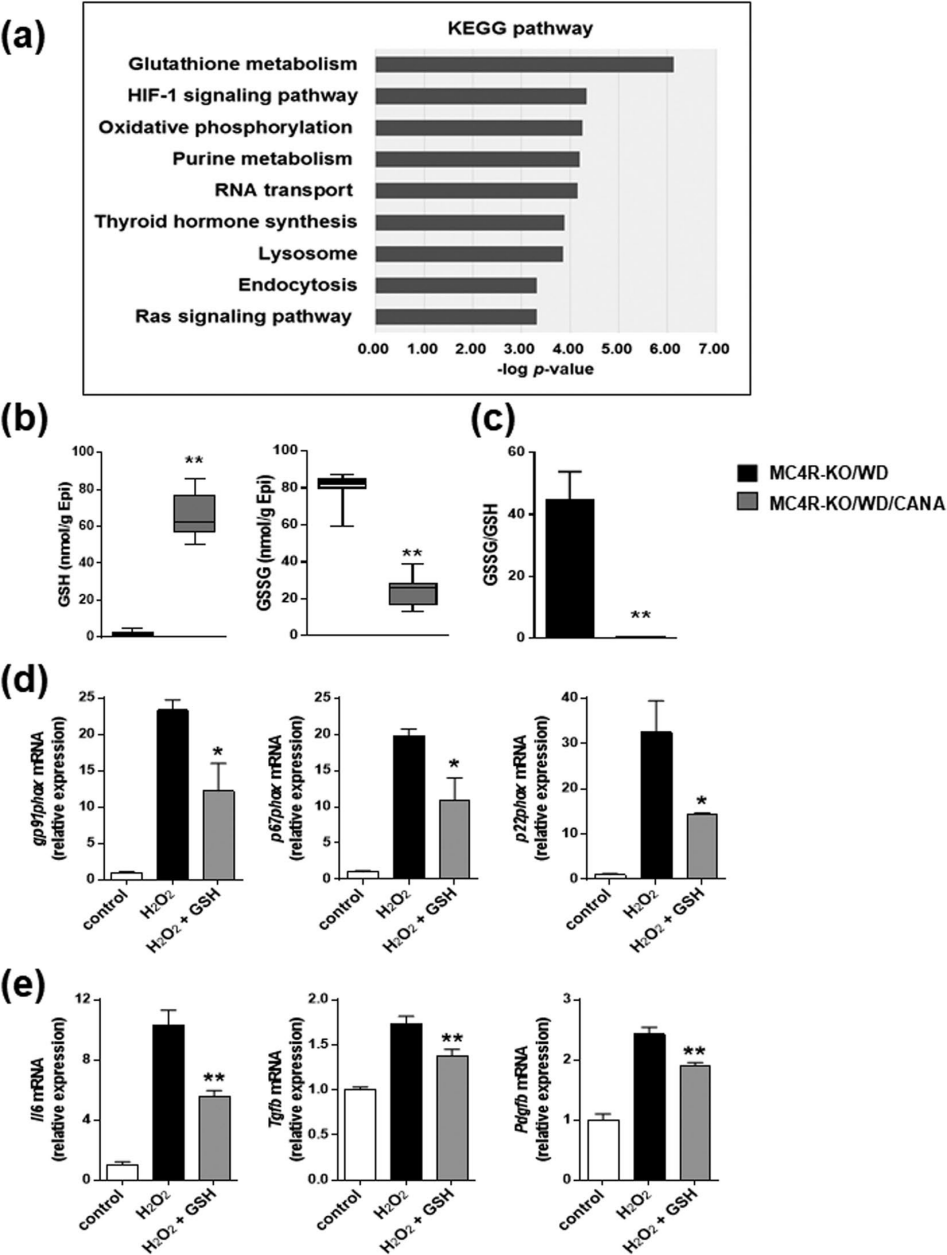
CANA increases GSH content in epididymal fat of WD-fed MC4R-KO mice. (**a**) The pathways enriched among the upregulated (>2.0-fold) metabolites in the epididymal fat of MC4R-KO mice treated with CANA for 8 weeks compared to those without treatment. The results are expressed as −log (*p* value). Metabolite concentrations of (**b**) GSH and GSSG, and (**c**) GSSG/GSH ratio of the epididymal fat. Data are mean ± SEM, *p < 0.05, **p < 0.01 vs MC4R-KO/WD; Student’s t test. *n* = 7. (**d**) Expression levels of NADPH oxidase complex genes in differentiated 3T3-L1 adipocytes treated with 200 µM H_2_O_2_ for 18 h after pretreatment with or without 1 mM GSH for 1 h. (**e**) Expression levels of inflammation-related genes in RAW 264.7 cells treated with 200 µM H_2_O_2_ for 18 h after pretreatment with or without 1 mM GSH for 1 h. *p < 0.05, **p < 0.01 vs H_2_O_2_. Statistical analyses were performed using Mann-Whitney (**b**), non-paired Student’s t (**c**), or one-way ANOVA (**d** and **e**) followed by Bonferroni post hoc test; H_2_O_2_ and H_2_O_2_ + GSH was pre-selected as a subset of means to compare. *n* = 4.

Recent evidence suggest that reduced lipid storage in obese adipose tissue contributes to ectopic lipid accumulation in non-adipose tissues such as the liver, skeletal muscle, and pancreas, where lipotoxicity impairs their metabolic functions^27^. To investigate the mechanisms by which CANA does not deteriorate inflammation or fibrosis in epididymal fat of WD-fed MC4R-KO mice, we performed metabolomic analysis of epididymal fat in MC4R-KO mice treated with CANA for eight weeks. Principal component analysis revealed that CANA had a substantial effect on the metabolite profile in epididymal fat (Supplementary Figure 5a), and that a pathway of glutathione metabolism was enriched as the most altered metabolic pathway by CANA (Fig. 4a). Whereas eight weeks of CANA treatment markedly increased the content of reduced glutathione (GSH) in the epididymal fat of WD-fed MC4R-KO mice (Fig. 4b), the content of oxidized glutathione (GSSG) was significantly reduced by CANA. As a result, CANA markedly suppressed the ratio of GSSG to GSH, an indicator of oxidative stress^28,29^, in WD-fed MC4R-KO mice (Fig. 4b). The ratio of GSSG to GSH in liver also showed a trend of decrease by CANA treatment (Supplementary Figure 5b). Whereas genes related to GSH metabolism such as *Cse*, *Cbs*, *Gclc*, and *Gclm* were unchanged by CANA treatment (data not shown), the induction of the NADPH oxidase complex genes in the epididymal adipose tissue of WD-fed MC4R-KO mice, such as *gp91phox*, *p22phox*, and *p67phox* were inhibited by CANA (Supplementary Figure 5c). Furthermore, pretreatment with GSH significantly attenuated the H_2_O_2_-induced upregulation of genes related to NADPH oxidase in 3T3-L1 adipocytes (Fig. 4c). As well, pretreatment with GSH significantly inhibited the H_2_O_2_-induced upregulation of *Il6*, *Tgfb*, and *Pdgfb* in RAW264.7 cells (Fig. 4d). Pretreatment with CANA also suppressed the H_2_O_2_-induced *Tgfb* upregulation in RAW264.7 cells (data not shown).

These data indicate that expanded adipose tissue in CANA-treated MC4R-KO mice is accompanied by an increase in GSH content and decrease in oxidative stress. Increased GSH may contribute to reduction in the H_2_O_2_-induced upregulation of NADPH oxidase in adipocytes, and inflammatory and fibrotic gene induction in macrophages.

### CANA attenuates the development to hepatocellular carcinoma in WD-fed MC4R-KO mice

**Figure 5:**
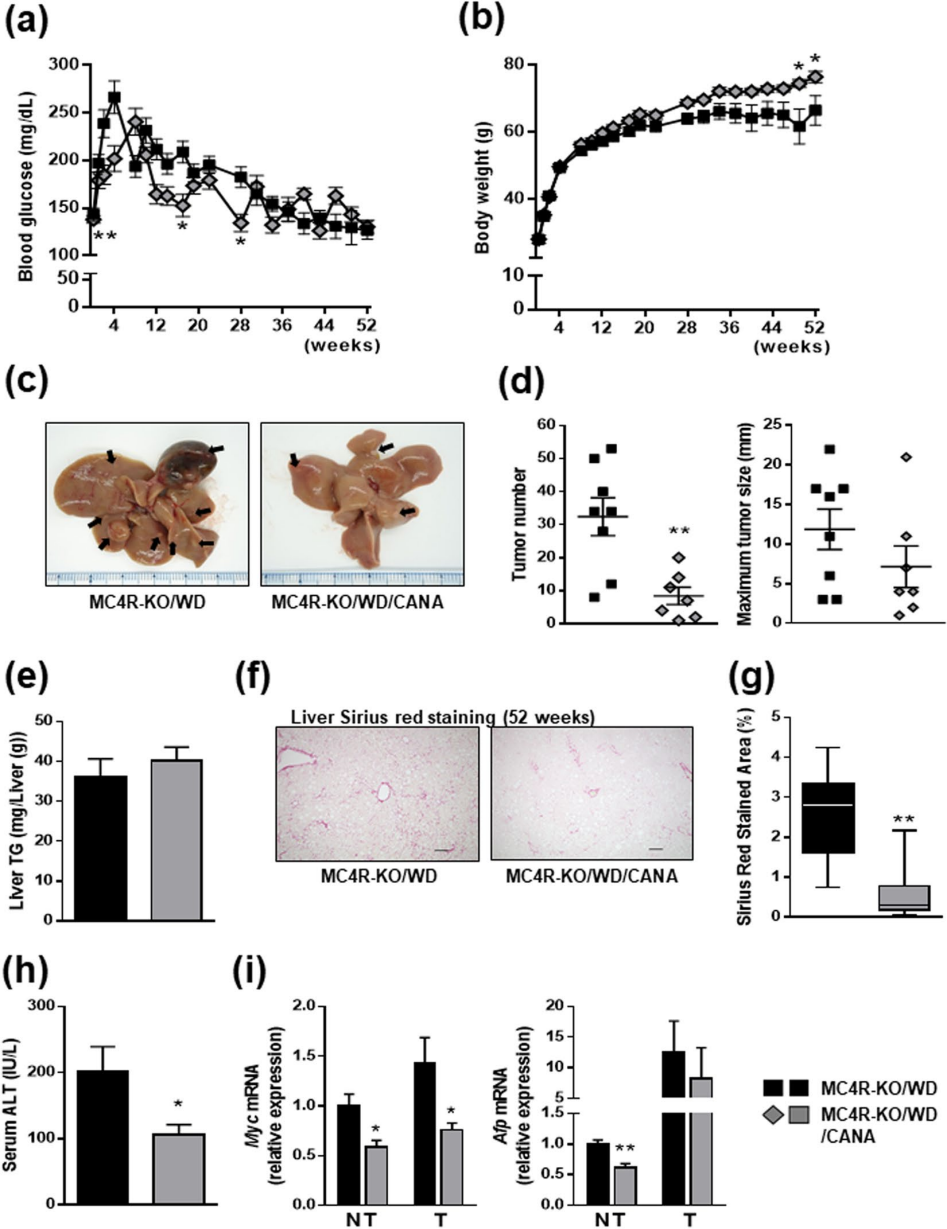
CANA attenuates the development to hepatocellular carcinoma in WD-fed MC4R-KO mice. The changes in (**a**) blood glucose levels and (**b**) body weight during CANA treatment for 52 weeks. *n* = 8–9. (**c**) Representative images of livers. Arrows indicate tumors. (**d**) Tumor numbers and maximum size. (**e**) Triglyceride content of the liver tissues from non-tumor sites. (**f**) Sirius red staining of the liver sections and (**g**) quantification. (**h**) Serum ALT levels. (**i**) Expression levels of *Myc* and *Afp* genes in the liver tissues from non-tumor (NT) or tumor (T) sites. Original magnification, ×100. Scale bars, 100 μm. *p < 0.05, **p < 0.01 vs MC4R-KO/WD. Statistical analyses were performed using non-paired Student’s t (**a**,**d**,**e**,**h** and **i**) or Mann-Whitney (**g**) test. *n* = 7–8.

We finally analyzed whether long-term treatment of CANA could prevent NASH-associated carcinogenesis in WD-fed MC4R-KO mice, which developed hepatocellular carcinoma after feeding on high-fat diet for one year^19^. During 52 weeks of treatment, a mouse from both CANA- and vehicle-treated groups died of liver tumors. Although their blood glucose profile during CANA treatment were not comparable to those on a 20-week CANA treatment protocol, possibly due to the differences in the method of glucose measurement and/or their breeding condition, CANA did suppress the glucose levels in WD-fed MC4R-KO mice (Fig. 5a). After 52 weeks of treatment CANA did increase the body weight change in WD-fed MC4R-KO mice (Fig. 5b), and all the animals from both groups developed liver tumors; however, 52-week CANA treatment significantly reduced the number of tumors in the liver of WD-fed MC4R-KO mice with a trend towards a reduction in maximum tumor size (Fig. 5c and d). Whereas CANA did not attenuate hepatic TG content in WD-fed MC4R-KO mice after treatment for 52 weeks (Fig. 5e), CANA significantly reduced liver fibrosis (Fig. 5f and g) and serum ALT levels (Fig. 5h). CANA treatment for 52 weeks also reduced expression of *Myc* and *Afp* in the tissues from tumor and/or non-tumor sites (Fig. 5i). CANA did not affect the serum insulin and hepatic Akt phosphorylation levels (Supplementary Figure 6a and b). These observations demonstrate that, along with hepatic steatosis and fibrosis, CANA can attenuate the progression to hepatocellular carcinoma in a mouse model of human NASH.

## Discussion

The present study demonstrated that CANA could effectively attenuate the development to hepatocellular carcinoma, as well as hepatic steatosis, inflammation, and fibrosis, in a mouse model of human NASH^19^. In addition to therapeutic effects of SGLT2 inhibitors on liver dysfunction, steatosis and/or steatohepatitis in humans^30–32^ and mice^11–17^, our observation suggests that SGLT2 inhibitors have preventive effects on a full spectrum of NAFLD in conditions of obesity, insulin resistance, and dyslipidemia. Our previous report showed that approximately 20% of patients treated with an SGLT2 inhibitor ipragliflozin for 24 weeks did not achieve more than 1% of body weight reduction^18^, probably due to enhanced appetite^33^. We therefore consider that CANA-treated mice reflect such T2DM patients with small or no body weight reduction by compensatory hyperphagia under treatment of SGLT2 inhibitors.

Based on the “multiple parallel hit” hypothesis, NAFLD pathogenesis is considered as a sequence of events from simple steatosis to hepatic inflammation toward fibrosis and NASH-associated hepatocellular carcinoma^34^. Whereas CANA suppressed hepatic TG accumulation at 8-week of CANA treatment, it did not at 20- and 52-week. It appears that CANA treatment delay or slow hepatic TG accumulation. Nonetheless, along to the “multiple parallel hit” hypothesis, the reduced hepatic TG accumulation by 8-week of CANA treatment could be considered to attenuate subsequent hepatic inflammation/fibrosis, and NASH-associated hepatocellular carcinoma as observed at 20- and 52-week of CANA treatment, respectively.

The adipose tissue can serve as energy storage reservoirs against calorie overload. Beyond its storage capacity, the excess calorie that is consumed results in ectopic fat accumulation in non-adipose tissues, such as the liver, skeletal muscle, and pancreas—where lipotoxicity leads to their metabolic dysfunctions and histopathologic changes^35^. Earlier we demonstrated that an SGLT2 inhibitor ipragliflozin promoted fat accumulation in the epididymal fat and prevented ectopic fat accumulation in the liver^18^; interestingly, the adipose expansion induced by ipragliflozin did not accompany an increase in inflammation or fibrosis, which may be referred to as “healthy adipose expansion”^36^. Correspondingly, CANA also attenuated hepatic steatosis in WD-fed MC4R-KO mice, after treatment for eight weeks, along with healthy adipose expansion, suggesting that the preventive effect on hepatic steatosis was due to the class effect(s) of SGLT2 inhibitors.

In addition to ectopic fat accumulation in the liver, *de novo* lipogenesis, which could be enhanced by hyperglycemia and hyperinsulinemia, is largely involved in inducing hepatic steatosis^37,38^. In the present study, as well as ipragliflozin^18^, 8 weeks of CANA treatment attenuated hyperglycemia and hyperinsulinemia, and suppressed the induction of genes involved in *de novo* lipogenesis of the liver. It therefore suggests that suppression of *de novo* lipogenesis, probably due to improved glucose homeostasis and insulin resistance, also contributes to attenuate hepatic steatosis in CANA-treated MC4R-KO mice. Considering low expression levels of SGLT2 (*Slc5a2*) in the liver and cellular component of the liver, CANA is unlikely to have direct effects on these cells to attenuate either hepatic steatosis or the subsequent pathologic changes.

As we previously reported^18^, one possible mechanism to promote healthy adipose expansion is improvement of insulin sensitivity in adipose tissue, which was assessed by Adipo-IR in the present study^23^. Based on the observation, the phenotypes of CANA-treated mice are similar to those of adipocyte-specific inducible phosphatase and tensin homolog (PTEN)-knockout mice, which exhibit enhanced adipocyte insulin signaling^39^; they gained more weight and adipose tissue during high-fat diet feeding, and showed enhanced insulin sensitivity, improved hepatic steatosis, and reduced adipose tissue inflammation. Thus, we consider that enhanced insulin sensitivity in adipose tissue can explain, at least partly, the increased lipid-storage capacity in adipocytes of CANA-treated mice. In addition, others and we have reported that fibrosis of white adipose tissue also limits its lipid-storage capacity via inhibiting adipocyte hypertrophy, which has a role in ectopic lipid accumulation in the liver^22,40^. It therefore suggests that reduced fibrosis of epididymal fat by CANA could additionally contribute to healthy adipose expansion in CANA-treated mice.

CANA markedly increased urinary glucose excretion in WD-fed MC4R-KO mice at 8 week of treatment; estimated from daily creatinine excretion of 24-week-old male C57BL/6j mice in a previous report^41^, daily glucose excretion at 8 week of CANA treatment was approximately 169 mg (0.68 kcal) in WD-fed MC4R-KO mice. Nonetheless, CANA did not reduce blood glucose levels of WD-fed MC4R-KO mice after treatment for 12 weeks in a 20-week treatment protocol. SGLT2 inhibitors have been shown to activate counter-regulatory responses that could compensate for its glucose-lowering effect; SGLT2 inhibitors increase plasma glucagon level to enhance endogenous glucose production in subjects with type 2 diabetes^42,43^, and enhance appetite in patients with T2DM^33^ and mice^18^. Given that CANA did not increase plasma glucagon levels in the present study, increased food intake may contribute to offsetting glucose-lowering effect of CANA after 12 weeks treatment. It is unclear as yet how SGLT2 inhibitors enhance appetite. It is assumed that the hyperphagia may represent an adaptive or compensatory response to involuntary glucose excretion, inasmuch as it occurs not immediately after the initiation of treatment with an SGLT2 inhibitor^33^. In recent years, there have been an increasing number of papers reporting that hypothalamic neurons have the capacity to sense fluctuations in local nutrient concentrations and to modify the activity in response to that sensation^44^. Taken together, the hyperphagia may be a consequence of physiological and adequate hypothalamic adaptations to changes in the energy sources, such as ketones and glucose, which vary according to the fasting state.

It is noteworthy that eight weeks of CANA treatment could significantly affect glutathione metabolism in the epididymal fat to decrease oxidative stress, as assessed by the ratio of GSSG to GSH. In 3T3-L1 adipocytes, hyperglycemia increases oxidative stress to cause inflammation and insulin resistance^45^. In erythrocytes from patients with type 2 diabetes, GSSG/GSH ratio were increased as compared to control subjects, which was reduced by insulin treatment *in vitro*^46,47^, suggesting that activation of insulin signaling decreases GSSG/GSH ratio. Although the precise mechanisms still remain unclear, improvement of both hyperglycemia and insulin sensitivity by CANA could contribute to decreasing GSSG/GSH ratio in adipose tissue.

Fat accumulation correlates with systemic oxidative stress in humans and mice, and ROS production was selectively increased in adipose tissue of obese mice, accompanied with augmented expression of NADPH oxidase^48^. Previous reports suggest that GSH could enhance adipose expansion, and inhibit inflammatory responses in adipocytes *in vitro*; buthionine-sulfoximine, a GSH inhibitor, inhibits adipogenesis in 3T3-L1 adipocytes^49^, and decreases adipose mass in mice^50^. Furthermore, hydrogen peroxide induces inflammatory responses in 3T3-L1 adipocytes^48^. In consistent with these reports, the present study demonstrated that GSH suppressed oxidative stress-induced upregulation of NADPH-oxidase, inflammatory responses, and fibrotic gene inductions in 3T3-L1 adipocytes. Taken together, it is conceivable that increase of GSH in adipose tissue of CANA-treated mice plays causal roles in adipose tissue expansion without deteriorating inflammation.

Whereas CANA suppressed NASH-associated hepatic carcinogenesis, it did not significantly reduce maximum tumor size. The observation is similar to a previous report using a thiazolidinedione; pioglitazone decreased the number of hepatic tumors in a mouse model for NASH, whereas it did not affect the maximum tumor size^51^. Of note, similar to CANA, thiazolidinediones have reported to attenuate hepatic steatosis, inflammation, and fibrosis associated with increased fat mass in human^7,52^. It therefore suggests that, at least in a mouse model of NASH, mechanisms of NASH-associated hepatic tumor growth may be distinct from those of carcinogenesis. Furthermore, agents that increase both insulin sensitivity and adiposity, like CANA and thiazolidinediones, may have preventive effects only on NASH-associated hepatic carcinogenesis.

Human observational studies have reported increased cancer mortality in those with obesity and T2DM, which may be at least in part explained by hyperinsulinemia, elevated insulin-like growth factor-1 (IGF-1), or potentially both^53–55^. However, because 20 and 52 week of CANA treatment did not affect serum insulin levels and/or Akt activity in the liver, improvement of hyperinsulinemia does not appear to be a main or direct mechanism to attenuate hepatocellular carcinogenesis in the present study. In addition, although the method of glucose measurement in a 52-week treatment protocol was different from that in 8- and 20-week treatment protocols, CANA did not attenuate hyperglycemia in WD-fed MC4R-KO throughout 52-week of WD feeding. It suggests that, even though it is assumed that CANA consistently enhances glucose excretion during entire 52 weeks, attenuation of hyperglycemia is also unlikely as a consistent factor to inhibit the development of hepatocellular carcinoma. Understanding the detailed molecular mechanisms by which CANA inhibits carcinogenesis, and generating clinical evidence to investigate whether SGLT2 inhibitors could prevent NASH-associated hepatocellular carcinoma in humans, require further studies.

In conclusion, CANA attenuated the development of hepatocellular carcinoma, hepatic steatosis, and fibrosis in a mouse model of NASH. The present study could provide new insight into the effects of SGLT2 inhibitors, not only as a glucose lowering agent, but also as a modulator of metabolic organ network, such as kidney, liver, adipose tissue, and possibly more. SGLT2 inhibitors can be considered as an optional treatment for T2DM patients with hepatic steatosis and NASH.

## Electronic supplementary material

Suppl Info
