# Supplementary material for: Canagliflozin, an SGLT2 inhibitor, attenuates the development of hepatocellular carcinoma in a mouse model of human NASH
Source: Sci Rep. 2018 Feb 5;8:2362. doi: 10.1038/s41598-018-19658-7 (PMC5799179; doi:10.1038/s41598-018-19658-7)
Supplement: Supplementary file 1 — Suppl Info [file 41598_2018_19658_MOESM1_ESM.pdf]

**Canagliflozin, an SGLT2 inhibitor, attenuates the development of hepatocellular carcinoma in a mouse model of human NASH**

Kumiko Shiba[1], Kyoichiro Tsuchiya[1, 2], Chikara Komiya[1], Yasutaka Miyachi[1], Kentaro Mori[1], Noriko Shimazu[1], Shinobu Yamaguchi[1], Naomi Ogasawara[1], Makoto Katoh[3], Michiko Itoh[4], Takayoshi Suganami[5], and Yoshihiro Ogawa[1, 6-8]

1) Department of Molecular Endocrinology and Metabolism, Graduate School of Medical and Dental Sciences, Tokyo Medical and Dental University, Tokyo, Japan; 2) Department of Diabetes, Yamanashi-Kosei Hospital, Yamanashi, Japan; 3) Medical Science Department Ikuyaku, Integrated Value Development Division, Mitsubishi Tanabe Pharma Corporation, Tokyo, Japan; 4) Department of Organ Network and Metabolism, Graduate School of Medical and Dental Sciences, Tokyo Medical and Dental University, Tokyo, Japan; 5) Department of Molecular Medicine and Metabolism, Research Institute of Environmental Medicine, Nagoya University, Nagoya, Japan; 6) Department of Molecular and Cellular Metabolism, Graduate School of Medical and Dental Sciences, Tokyo Medical and Dental University, Tokyo, Japan; 7) Department of Medical and Bioregulatory Science, Graduate School of Medical Sciences, Kyushu University, Fukuoka, Japan; 8) Japan Agency for Medical Research and Development, CREST, Tokyo, Japan

<Correspondence >

K.T. (ktsuchiya-dm@umin.ac.jp) and Y.O. (ogawa.mem@tmd.ac.jp). Department of Molecular Endocrinology and Metabolism, Graduate School of Medical and Dental Sciences, Tokyo Medical and Dental University. 1-5-45, Yushima, Bunkyo-ku, Tokyo, 113-8510, Japan. Tel: +81-3-5803-5216, Fax: +81-3-5803-0172

# Supplementary Figure 1

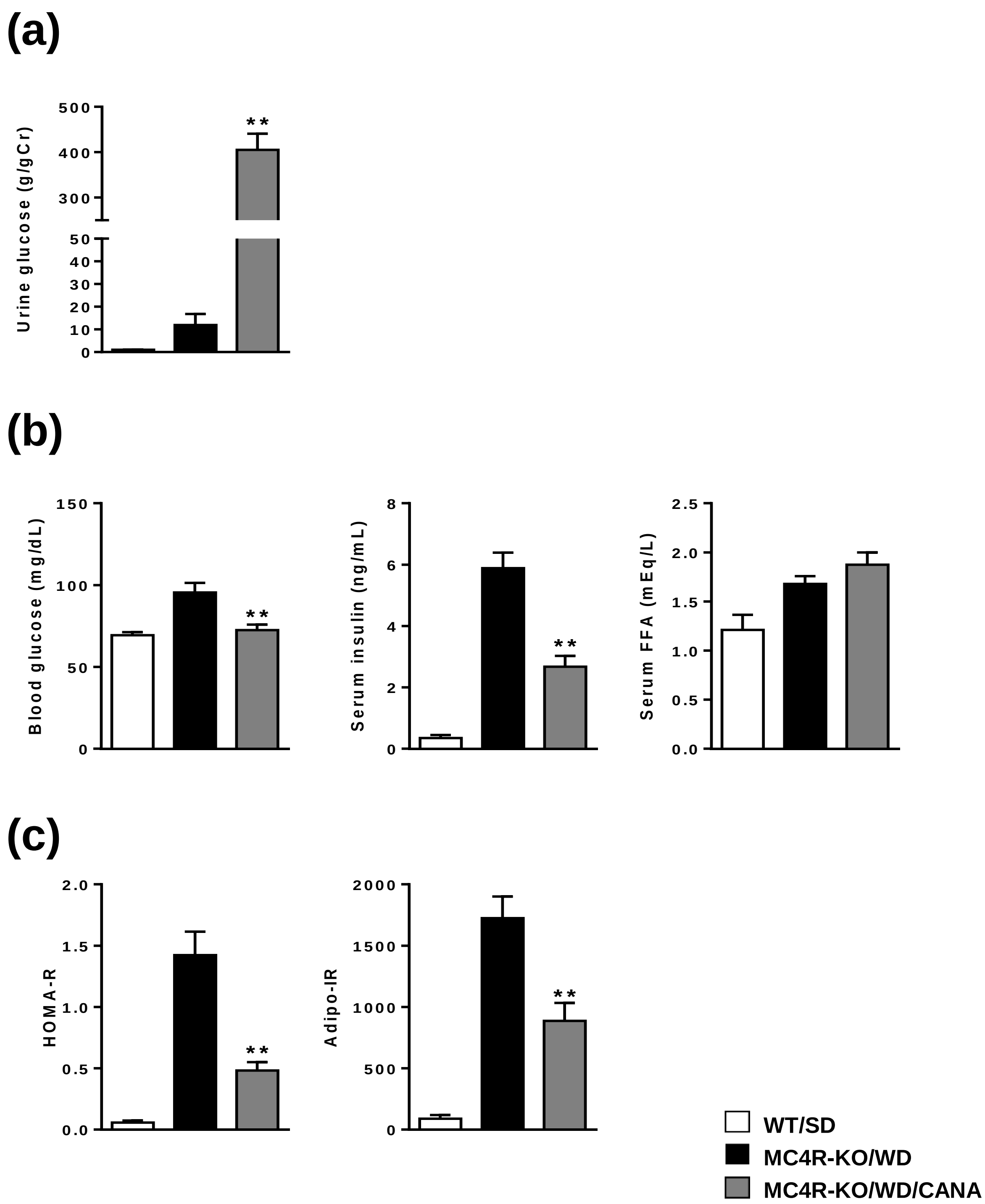

# Supplementary Figure 2

## (a)

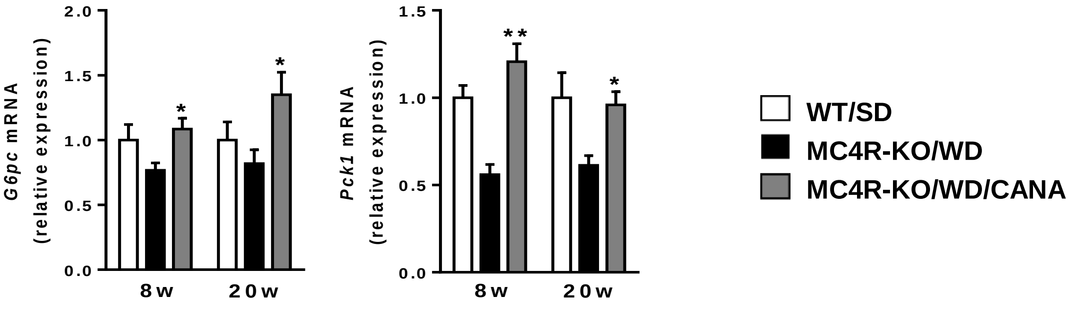


## (b)

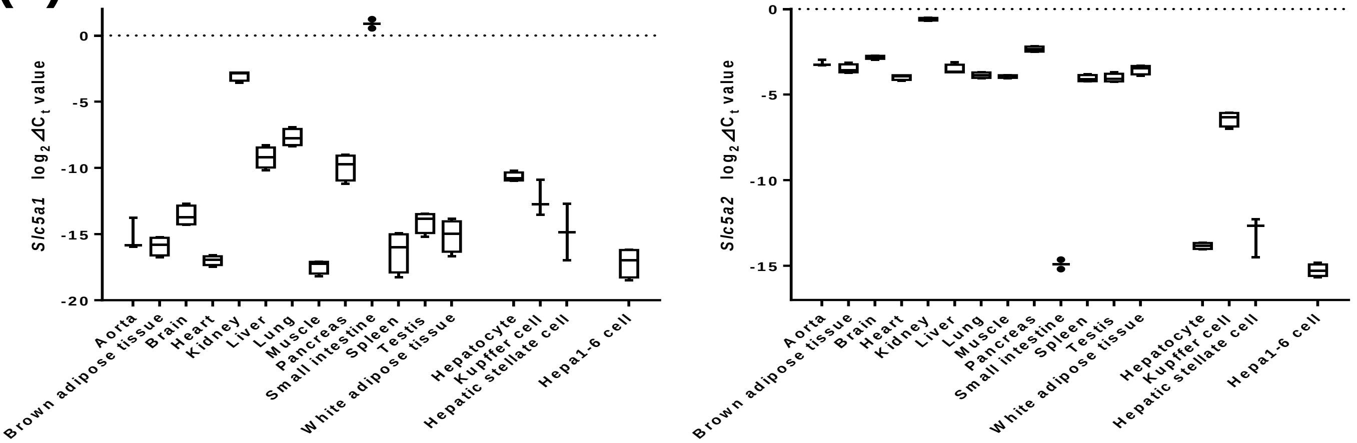


## (c)

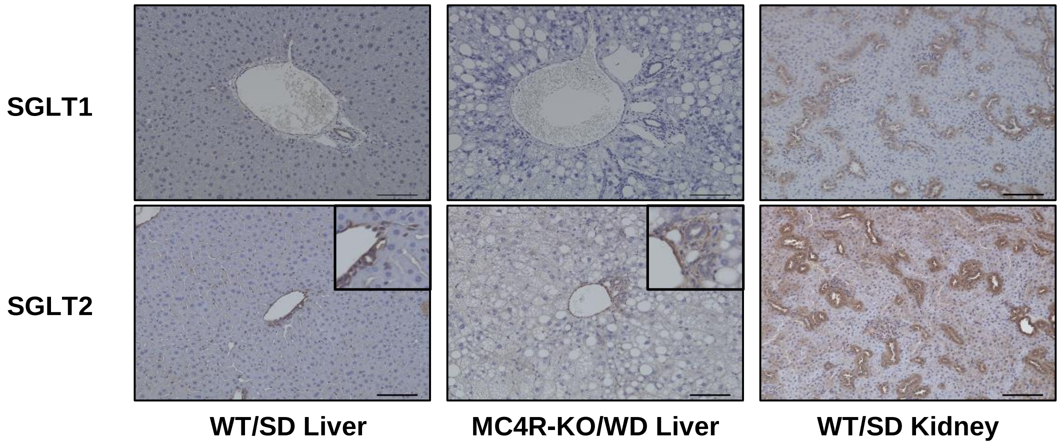

# Supplementary Figure 3

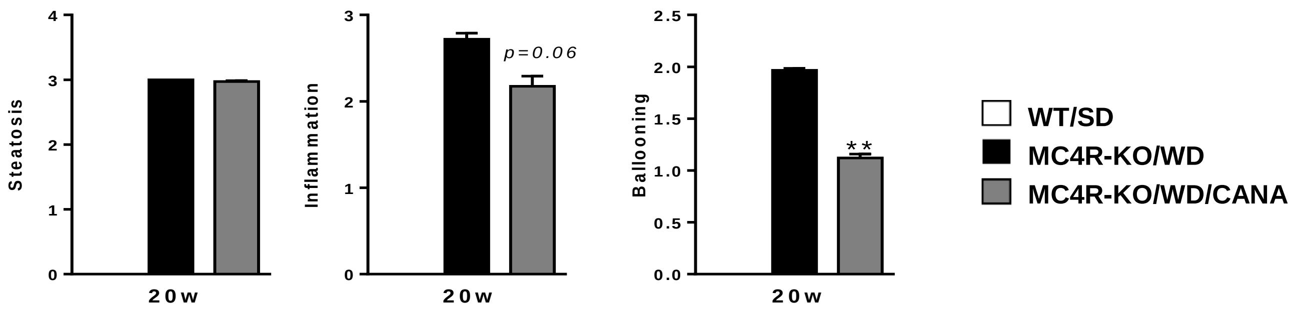

# Supplementary Figure 4

## (a)

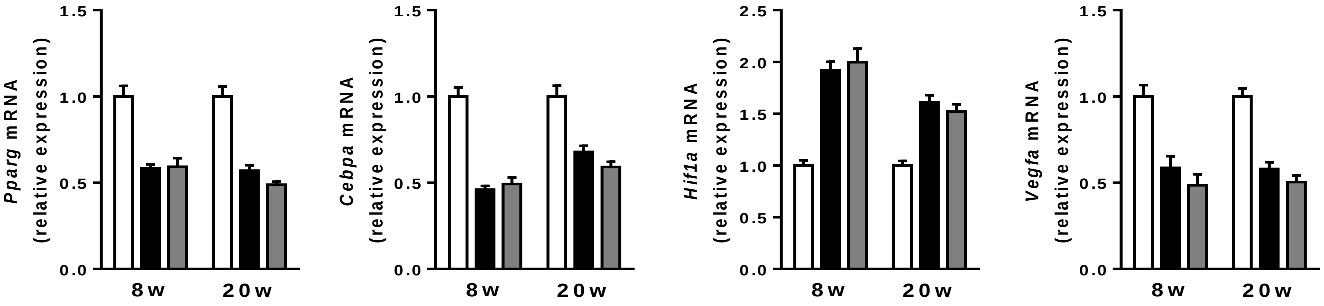


## (b)

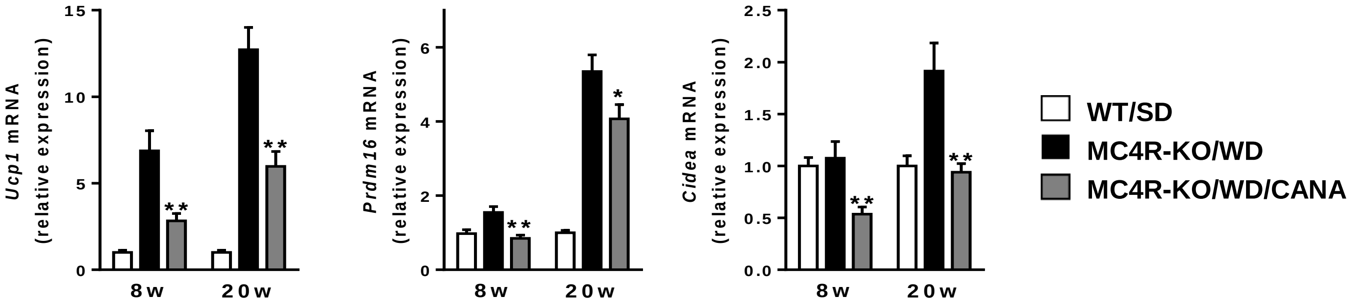

# Supplementary Figure 5

(a)

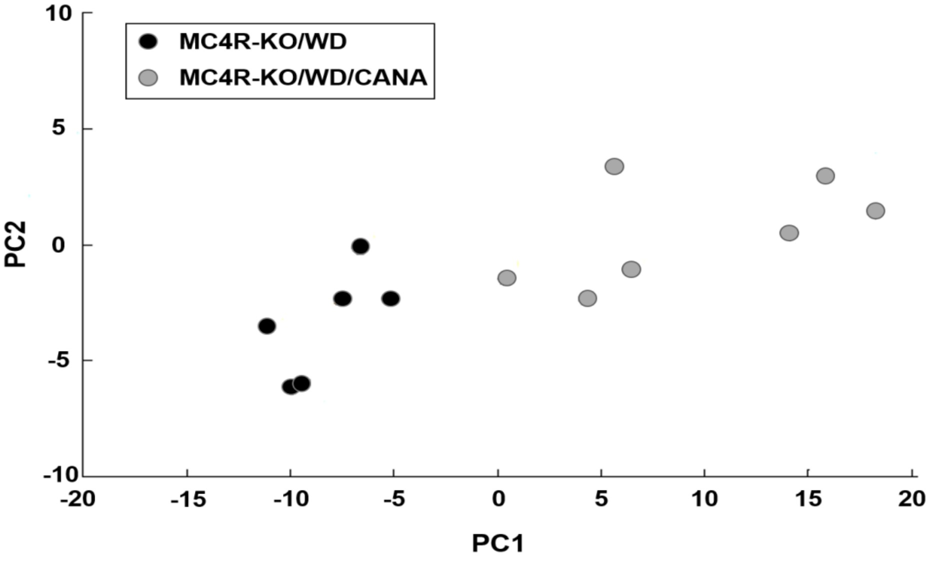


(b)

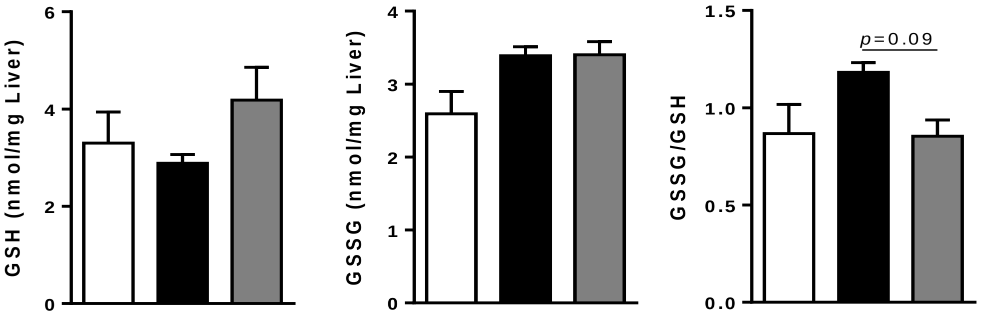


(c)

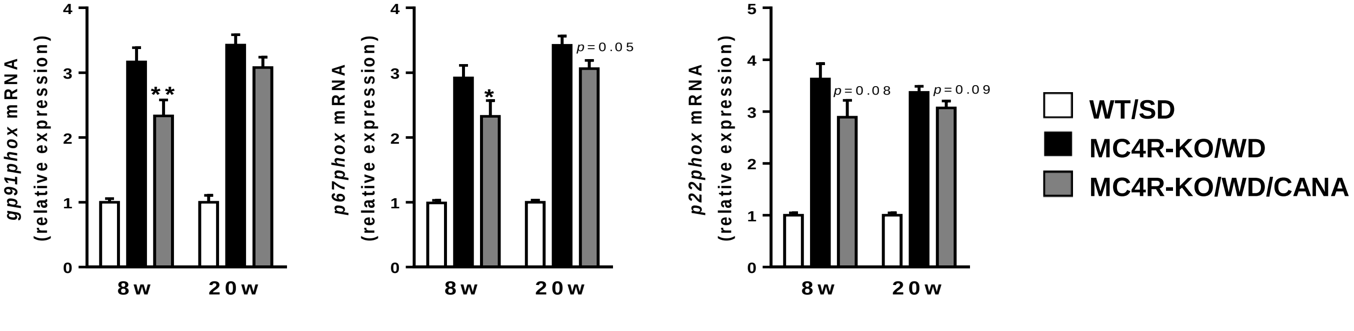

# Supplementary Figure 6

(a)

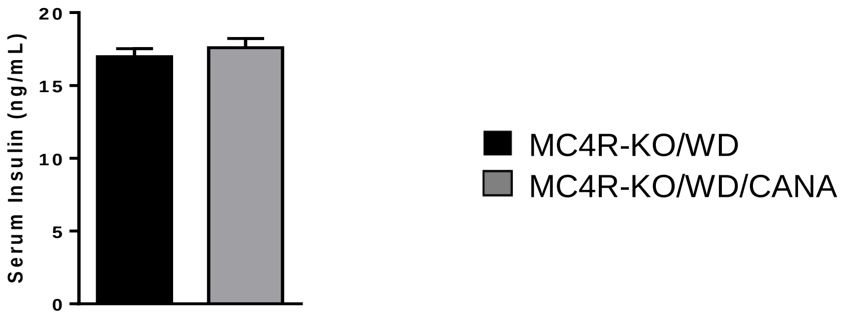


(b)

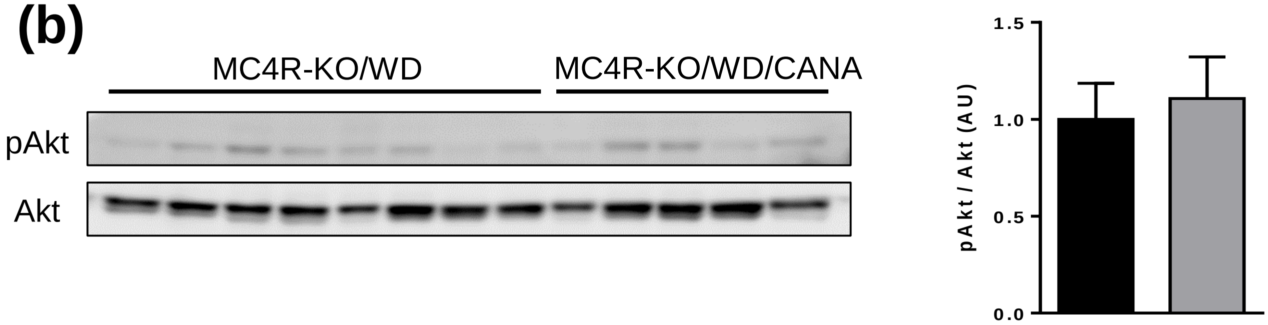

# Supplementary Figure 7

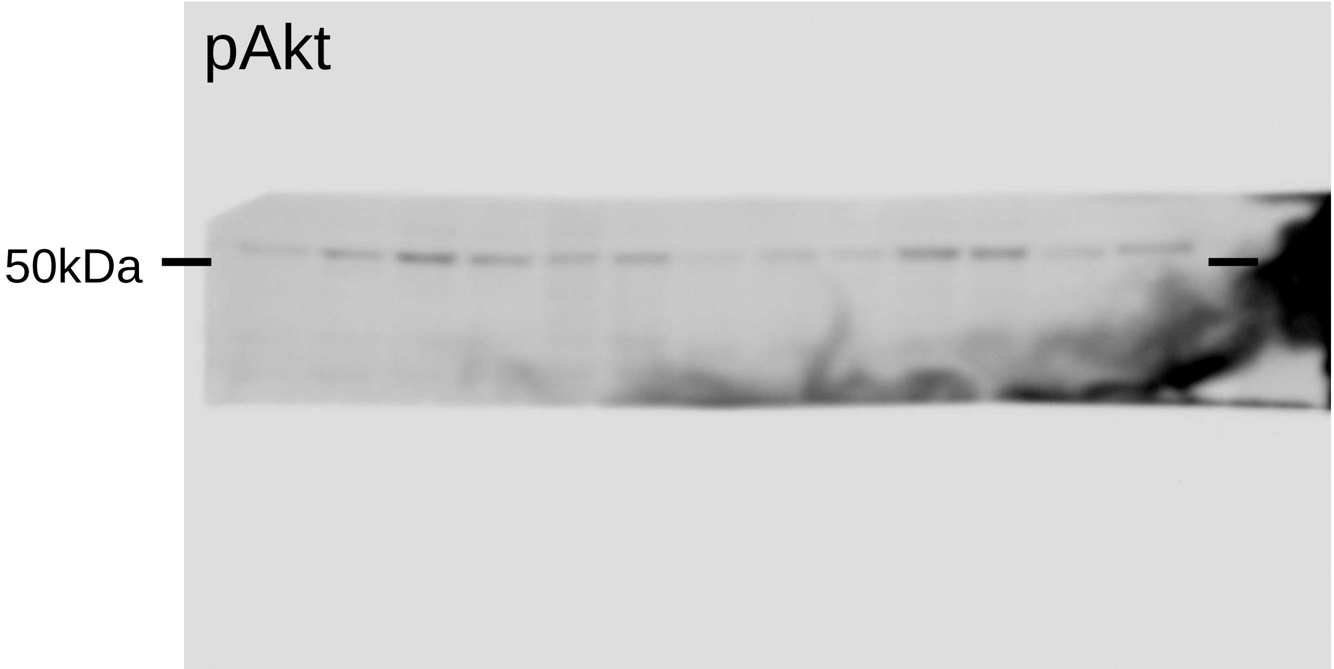


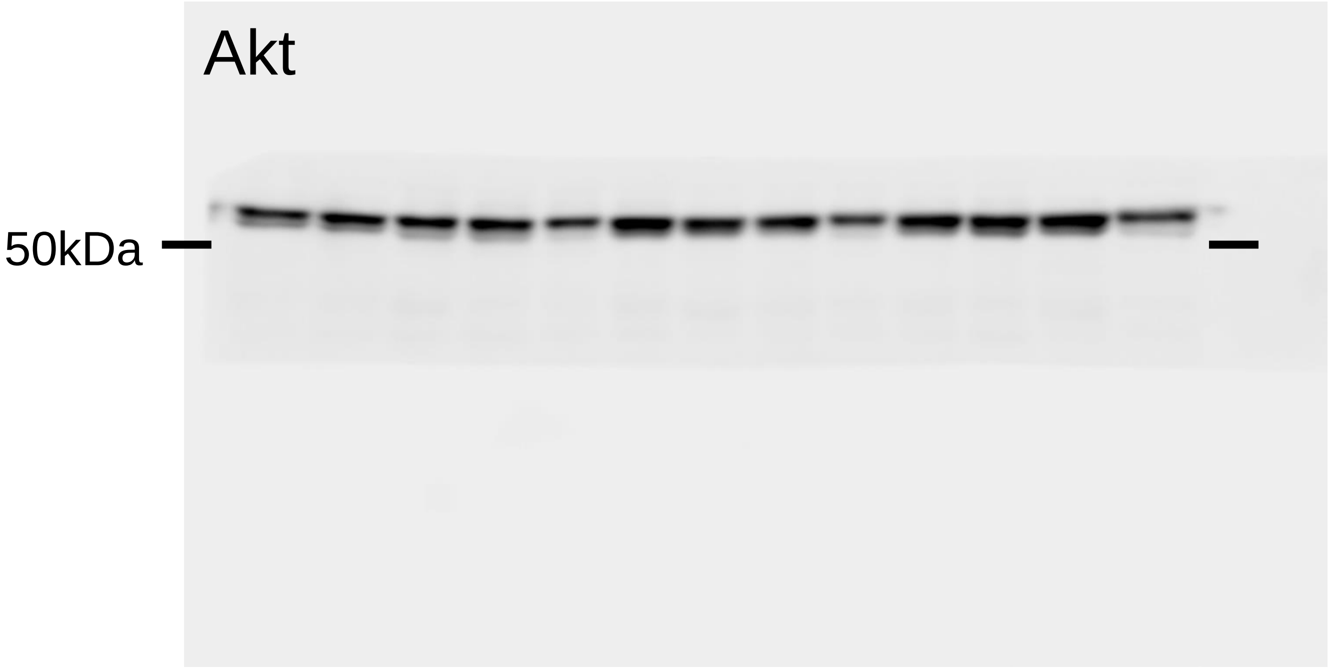

## SUPPLEMENTARY FIGURE LEGENDS

**Supplementary Figure 1. Effects of 8 weeks of CANA treatment on urine glucose excretion and insulin sensitivity.**

(a) Urine glucose excretion, and (b) fasting glucose, serum insulin, and free-fatty acid (FFA) levels after 8 weeks of CANA treatment. (c) HOMA-R and Adipo-IR indices after 8 weeks of CANA treatment. ** $p < 0.01$ vs MC4R-KO/WD. Statistical analyses were performed using one-way ANOVA followed by Bonferroni post hoc test; MC4R-KO/WD and MC4R-KO/WD/CANA are a pre-selected subset of means to compare.

**Supplementary Figure 2. Expression of gluconeogenesis-related genes in the liver after CANA treatment and SGLT1 and SGLT2 expression in various tissues**

(a) Eight-week-old MC4R-KO mice were fed WD for 8 and 20 weeks. CANA was mixed with WD. Control WT mice fed SD. qPCR analysis of gluconeogenesis genes in liver. * $p < 0.05$, ** $p < 0.01$ vs MC4R-KO/WD. Statistical analyses were performed using one-way ANOVA followed by Bonferroni post hoc test; MC4R-KO/WD and MC4R-KO/WD/CANA are a pre-selected subset of means to compare. $n = 15$. (b) Expression of *Slc5a1* and *Slc5a2* mRNAs in 8-week old wild-type mice tissues, liver cells, and Hepa1-6 cell lines. $n = 2$–4. (c) SGLT1 and SGLT2 immunohistochemical analysis of the liver and kidney.

**Supplementary Figure 3. Effect of 20 weeks of CANA treatment on liver histology.**

The histological scores of steatosis, inflammation, and ballooning in the liver after 20 weeks of CANA treatment. * $p < 0.05$, ** $p < 0.01$ vs MC4R-KO/WD. Statistical analyses were performed using one-way ANOVA followed by Bonferroni post hoc test; MC4R-KO/WD and MC4R-KO/WD/CANA are a pre-selected subset of means to compare. $n = 15$.

**Supplementary Figure 4. Gene expression in epididymal adipose tissue.**

Expression levels of (a) adipogenesis-related and (b) brown adipocyte-selective genes in epididymal fat. * $p < 0.05$, ** $p < 0.01$ vs MC4R-KO/WD. Statistical analyses were performed using one-way ANOVA followed by Bonferroni post hoc test; MC4R-KO/WD and MC4R-KO/WD/CANA are a pre-selected subset of means to compare. $n = 15$.

**Supplementary Figure 5. Principal component analysis of metabolites and gene expression in epididymal adipose tissue.**

(a) Principal component analysis of metabolites in the epididymal fat of MC4R-KO mice after 8 weeks of CANA treatment. Score plots for PC1 and PC2 are shown. $n = 7$. (b) qPCR analysis of NADPH oxidase complex genes in epididymal fat. * $p < 0.05$, ** $p < 0.01$ vs MC4R-KO/WD. Statistical analyses were performed using one-way ANOVA followed by Bonferroni post hoc test; MC4R-KO/WD and MC4R-KO/WD/CANA are a pre-selected subset of means to compare. $n = 15$.

**Supplementary Figure 6. Effect of 52 weeks of CANA treatment on serum insulin levels and the liver.**

Eight-week-old MC4R-KO mice were fed WD for 52 weeks. CANA was mixed with WD. (a) Serum insulin levels. (b) Immunoblots and quantification of phosphorylated Akt (S473) in liver tumor tissues. Full blots are given in the Supplementary Figure 7. Statistical analyses were performed using Student's *t* test. $n = 5–8$.

**Supplementary Figure 7. Full blots of phosphorylated Akt (pAkt) and Akt in Supplementary Figure 6**

## SUPPLEMENTARY METHODS

### Biochemical assays

Serum insulin and glucagon using Mouse Insulin ELISA Kit (Morinaga Institute of Biological Science, Inc., Kanagawa, Japan) and Mercodia Glucagon ELISA kit (Mercodia AB, Uppsala, Sweden), respectively; Total GLP-1 and active GLP-1 using Multi Species GLP-1 Total ELISA (EZGLP1T-36K, Millipore, Corp., Billerica, MA, USA) and Rodent Active GLP-1 (7-36) ELISA Kit (KT878, Epitope Diagnostics, Inc., San Diego, CA, USA), respectively; Serum alanine aminotransferase (ALT) using Fuji Dry-chem 7000V (Fujifilm corporation, Tokyo, Japan); and liver TG content using TG E-Test Wako. The total lipids were extracted from the liver using chloroform and methanol (2:1 v/v).

### Cell isolation

Primary hepatocytes and Kupffer cells (KC) were isolated from liver segments obtained from male C57BL/6J mice and sorted the primary hepatocytes and KC into QIAzol Lysis Reagent (QIAGEN, Inc., Valencia, CA, USA) on a FACSAriaII (BD Biosciences) as previously described [1]. Hepatic stellate cells were isolated using collagenase perfusion as previously described [2].

### Histological analysis

Upon sacrifice after 52 weeks of CANA treatment, we counted the visible liver tumors (>1 mm) and measured their size. The paraformaldehyde-fixed liver and epididymal fat were embedded in paraffin. Liver sections were stained using hematoxylin and eosin (HE) for the assessment of NAFLD activity score (NAS) [3]. Liver and epididymal fat were stained using Sirius red for the assessment of fibrosis; positive areas were measured using NIH Image J software. Adipocyte size was quantified with the open-share Fiji program Adiposoft 1.13 (http://fiji.sc/Adiposoft). Macrophages in the liver and epididymal fat were immunohistochemically detected using a rat monoclonal F4/80 antibody (MCA497GA; 1:1000 dilution; AbD Serotec Ltd., Kidlington, UK). The number of crown-like structures (CLS) and hepatic CLS (hCLS) were quantified by counting their total numbers in each section and expressed as the mean per square millimeter. Immunohistochemical staining of SGLT1 and

SGLT2 in the liver and kidney were performed using anti-SGLT1 rabbit polyclonal antibody (ab14686, 1:100 dilution; Abcam, Ltd., Cambridge, UK) and anti-SGLT2 rabbit polyclonal antibody (ab37296; 1:200 dilution; Abcam, Ltd., Cambridge, UK), respectively. Binding of primary antibody was visualized using DAB+ chromogen (Dako, Glostrup, Denmark). All microscopic images were acquired using the Keyence BZ-9000 microscope.

**Metabolomic analysis of epididymal adipose tissue**

Approximately 350 mg of frozen tissue was plunged into 1 ml of 50% acetonitrile/Milli-Q water containing internal standards (Solution ID: 304-1002, Human Metabolome Technologies, Inc., Tsuruoka, Japan) at 0 ºC to inactivate the enzymes. The tissue was homogenized at 1,100 rpm for 120 s using a tissue homogenizer and then the homogenate was centrifuged at 2,300 × g and 4 ºC for 5 min. Subsequently, 400 μl of the upper aqueous layer was centrifugally filtered through a HMT 5-kDa cutoff filter at 9,100 × g and 4 ºC for 120 min to remove proteins. The filtrate was centrifugally concentrated and re-suspended in 50 μl of Milli-Q water for CE-MS analysis. Extracted metabolites were analyzed by Agilent CE-TOFMS system (Agilent Technologies, Waldbronn, Germany). Peaks were extracted using automatic integration software MasterHands ver.2.17.1.11 (Keio University, Tsuruoka, Japan) in order to obtain peak information including m/z, migration time for CE-TOFMS measurement (MT), and peak area. Concentrations of metabolites were calculated by normalizing the peak area of each metabolite with respect to that of the internal standard. A standard curve for each metabolite was obtained by single-point analysis of 100 μM standard metabolites. Hierarchical cluster analysis (HCA) and principal component analysis (PCA) were performed using HMT's proprietary software PeakStat and SampleStat, respectively. A pathway analysis of metabolic profiles was performed with ConsensusPathDB.

**Measurement for GSH and GSSG content in liver**

GSH and GSSG content in liver were measured using Bioxytech GSH/GSSG-412 kit (Oxis Research, Portland, OR) according to the manufacturer's instructions. Liver tissue sample were homogenized in a 5% metaphosphoric acid. After homogenization, they were centrifuged and the supernatant was used to measure the concentrations of GSH and GSSG.

**Supplementary Table 1. List of primers.**

| | | |
|---|---|---|
| *36b4* | Fw | GGCCCTGCACTCTCGCTTTC |
| | Rv | TGCCAGGACGCGCTTGT |
| *Acc1* | Fw | TGAGATTGGCATGGTAGCCTG |
| | Rv | CTCGGCCATCTGGATATTCAG |
| *Fasn* | Fw | CCTGGATAGCATTCCGAACCT |
| | Rv | AGCACATCTCGAAGGCTACACA |
| *Scd1* | Fw | ACGCCGACCCTCACAATTC |
| | Rv | CAGTTTTCCGCCCTTCTCTTT |
| *Emr1* | Fw | CTTTGGCTATGGGCTTCCAGTC |
| | Rv | GCAAGGAGGACAGAGTTTATCGTG |
| *Cd11c* | Fw | GCCATTGAGGGCACAGAGA |
| | Rv | GAAGCCCTCCTGGGACATCT |
| *Tnfa* | Fw | ACCCTCACACTCAGATCATCTTC |
| | Rv | TGGTGGTTTGCTACGACGT |
| *Col1a1* | Fw | CCTCAGGGTATTGCTGGACAAC |
| | Rv | ACCACTTGATCCAGAAGGACCTT |
| *Acta2* | Fw | AGCCATCTTTCATTGGGATGG |
| | Rv | CCCCTGACAGGACGTTGTTA |
| *Tgfb* | Fw | CCTGAGTGGCTGTCTTTTGACG |
| | Rv | AGTGAGCGCTGAATCGAAAGC |
| *Timp1* | Fw | CATCACGGGCCGCCTA |
| | Rv | AAGCTGCAGGCACTGATGTG |
| *Col6a1* | Fw | GATGAGGGTGAAGTGGGAGA |
| | Rv | CAGCACGAAGAGGATGTCAA |
| *Col6a3* | Fw | CAGAACCATTGTTTCTCACT |
| | Rv | AGGACTACACATCTTTTCAC |
| *gp91phox* | Fw | CATTGTCACCGATGTCAGAGAGA |
| | Rv | GTCGGGATTTCTGACCGGTAT |
| *p67phox* | Fw | AAGCAAAAAGAGCCCAAGGAA |
| | Rv | CATGTAAGGCATAGGCACGCT |

| | | |
|---|---|---|
| *p22phox* | Fw | CATGGAGCGATGTGGACAGA |
| | Rv | CCCGAAAAGCTTCACCACAG |
| *Il6* | Fw | ACAACCACGGCCTTCCCTACTT |
| | Rv | CACGATTTCCCAGAGAACATGTG |
| *Pdgfb* | Fw | CCATCCGCTCCTTTGATGAT |
| | Rv | TCAGCCCCATCTTCATCTACG |
| *Myc* | Fw | TGACCTAACTCGAGGAGGAGCTGGAATC |
| | Rv | AAGTTTGAGGCAGTTAAAATTATGGCTGAAGC |
| *Afp* | Fw | TGGTTACACGAGGAAAGCCC |
| | Rv | AATGTCGGCCATTCCCTCAC |
| *Slc5a1* | Fw | GCCATGGGTGGCTTTGAATG |
| | Rv | GAGCAGGGACAGAACGGAAA |
| *Slc5a2* | Fw | GCTGCCTATTTCCTGCTGGT |
| | Rv | GAACAGAGAGGCTCCAACCG |
| *G6pc* | Fw | CACCTGTGAGACCGGACCA |
| | Rv | GACCATAACATAGTATACACCTGCTGC |
| *Pck1* | Fw | CCACAGCTGCTGCAGAACA |
| | Rv | GAAGGGTCGCATGGCAAA |
| *Sod1* | Fw | CAGCATGGGTTCCACGTCCA |
| | Rv | CACATTGGCCACACCGTCCT |
| *Catalase* | Fw | CTCGCAGAGACCTGATGTCC |
| | Rv | GACCCCGCGGTCATGATATT |
| *Hif1a* | Fw | CAAGATCTCGGCGAAGCA |
| | Rv | GGTGAGCCTCATAACAGAAGCTTT |
| *Vegfa* | Fw | GGAGACTCTTCGAGGAGCACTT |
| | Rv | GGCGATTTAGCAGCAGATATAAGAA |
| *Pparg* | Fw | ACAATGCCATCAGGTTTGGG |
| | Rv | CCGCCAACAGCTTCTCCTT |
| *Cebpa* | Fw | CGCCTTCAACGACGAGTTC |
| | Rv | TTGGCCTTCTCCTGCTGTC |
| *Ucp1* | Fw | CGCTGGACACTGCCAAAGT |

| | | |
|---|---|---|
| | Rv | GGTGGTGATGGTCCCTAGGA |
| *Prdm16* | Fw | CAGCACGGTGAAGCCATTC |
| | Rv | GCGTGCATCCGCTTGTG |
| *Cidea* | Fw | GACAGAAATGGACACCGGGTA |
| | Rv | TGTGCATCGGATGTCGTAGG |
| *Pgc1a* | Fw | CGATGTGTCGCCTTCTTGCT |
| | Rv | CGAGAGCGCATCCTTTGG |

**REFERENCES**


1 Miyachi, Y. *et al.* Roles for Cell-Cell Adhesion and Contact in Obesity-Induced Hepatic Myeloid Cell Accumulation and Glucose Intolerance. *Cell reports* **18**, 2766-2779, doi:10.1016/j.celrep.2017.02.039 (2017).

2 Mederacke, I., Dapito, D. H., Affo, S., Uchinami, H. & Schwabe, R. F. High-yield and high-purity isolation of hepatic stellate cells from normal and fibrotic mouse livers. *Nat Protoc* **10**, 305-315, doi:10.1038/nprot.2015.017 (2015).

3 Kleiner, D. E. *et al.* Design and validation of a histological scoring system for nonalcoholic fatty liver disease. *Hepatology* **41**, 1313-1321, doi:10.1002/hep.20701 (2005).
